# Genotype‐dependent and heat‐induced grain chalkiness in rice correlates with the expression patterns of starch biosynthesis genes

**DOI:** 10.1002/pei3.10054

**Published:** 2021-06-15

**Authors:** Peter James Gann, Manuel Esguerra, Paul Allen Counce, Vibha Srivastava

**Affiliations:** ^1^ Cell and Molecular Biology Program University of Arkansas Fayetteville AR USA; ^2^ Department of Crop, Soil and Environmental Sciences University of Arkansas Fayetteville AR USA; ^3^ Rice Research and Extension Center Stuttgart AR USA; ^4^ Department of Horticulture University of Arkansas Fayetteville AR USA

**Keywords:** chalkiness, heat stress, high nighttime temperature, rice grain, starch biosynthesis

## Abstract

Starch biosynthesis is a complex process underlying grain chalkiness in rice in a genotype‐dependent manner. Coordinated expression of starch biosynthesis genes is important for producing translucent rice grains, while disruption in this process leads to opaque or chalky grains. To better understand the dynamics of starch biosynthesis genes in grain chalkiness, six rice genotypes showing variable chalk levels were subjected to gene expression analysis during reproductive stages. In the chalky genotypes, peak expression of the large subunit genes of *ADP‐glucose* *pyrophosphorylase* (*AGPase*), encoding the first key step in starch biosynthesis, occurred in the stages before grain filling commenced, creating a gap with the upregulation of starch synthase genes, *granule bound starch synthase I* (*GBSSI*) and *starch synthase IIA* (*SSIIA*). Whereas, in low‐chalk genotypes, *AGPase* large subunit genes expressed at later stages, generally following the expression patterns of *GBSSI* and *SSIIA*. However, heat treatment altered the expression in a genotype‐dependent manner that was accompanied by transformed grain morphology and increased chalkiness. The suppression of *AGPase* subunit genes during early grain filling stages was observed in the chalky genotypes or upon heat treatment, which could result in a limited pool of ADP‐Glucose for synthesizing amylose and amylopectin, the major components of the starch. This suboptimal starch biosynthesis process could subsequently lead to inefficient grain filling and air pockets that contribute to chalkiness. In summary, this study suggests a mechanism of grain chalkiness based on the expression patterns of the starch biosynthesis genes in rice.

## Graphical abstract

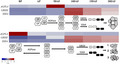

## 1 INTRODUCTION

Physical, granular, and chemical properties are the measures of grain quality, which are dependent on the starch biosynthesis process. Grain chalkiness is a highly undesirable trait in rice, which is genotype‐dependent and can also be induced by the high nighttime temperature (HNT) among other factors (Feng et al., 2017; Jagdish et al., 2015; Lanning et al., 2011; Xu et al., 2020). Coordination of the enzymes involved in this process is important to prevent grain chalk that affects the market value, cooking, and eating quality of the rice (Fitzgerald & Resurreccion, 2009; Lisle et al., 2000).

Starch biosynthesis in the developing endosperms of cereal grains is a complex process recently reviewed by Tetlow and Emes (2017). Briefly, starch biosynthesis starts after fertilization, when the endosperm cells multiply, form cell walls, and elongate. Formation and elongation of cell walls utilizes imported sucrose that is converted to glucose and fructose by cell wall invertase (Wang et al., 2008). These hexose sugars are then transported into endosperm cells, and subsequently, converted to Glucose‐1‐Phosphate through the action of several enzymes in the cytosol, and ultimately converted to ADP‐glucose by ADP‐glucose pyrophosphorylase (AGPase), the first key enzyme in the starch biosynthesis pathway. ADP‐glucose is transported into amyloplast to serve as the substrate for starch synthases filling the grain with storage starch, an important compound in grain physical quality. AGPase catalyzed reaction is the rate‐limiting step in the process (Stark et al., 1992), and with the reversibility of this reaction, the follow‐up expression of *granule bound starch synthases* (*GBSSs*) and *starch synthases* (*SS*) is important to utilize ADP‐glucose and prevent the futile cycle of converting ADP‐glucose to glucose‐1‐phosphate.

Granule bound starch synthase synthesizes amylose with *α* (1→4) glycoside linkage, and SS elongates polysaccharide chain with *α* (1→4 and 1→6) glycoside linkages synthesizing amylopectin. *Granule bound starch synthase I* (*GBSSI*) and *starch synthase IIA* (*SSIIA*) are the highly expressed isoforms in the rice endosperm during grain filling stages (Hirose & Terao, 2004; Ohdan et al., 2005; Umemoto & Terashima, 2002; Xing et al., 2016). Mutations in starch synthase genes reportedly affect grain chalkiness by altering the granule morphology from compound polyhedral type to simple spherical type (Kusano et al., 2012; Toyosawa et al., 2016). The simple, spherical granules constitute the chalk portion as they pack loosely and include airspaces (Kaneko et al., 2016; Kim et al., 2004; Lu et al., 2015; Mitsui et al., 2016). Gene expression profiling of chalky and translucent grains in Japanese rice showed genotypic and heat‐induced changes in the starch genes. Specifically, starch synthesis genes were found to be upregulated in a near isogenic line showing high chalkiness in comparison to its normal parent (Liu et al., 2010). However, transcriptomics of Nipponbare caryopses ripened in heat or normal temperature showed suppression of starch synthesis genes in heat, even though, heat treatment‐induced chalkiness in the grains (Yamakawa et al., 2007). Thus, starch synthesis genes play a major role in determining grain quality; however, their coordination with one another and expression patterns related to chalky or translucent grains has not been fully understood.

Other mechanisms that control grain chalkiness are related to starch accumulation and degradation processes. For example, disruption of the amyloplast's outer envelope membrane during seed maturation leads to the abundance of simple and spherical granules (Toyosawa et al., 2016), and early degradation of starch through amylase activity contributes to grain chalkiness. Micropores on the surfaces of rough amyloplast in the chalky grains indicate starch degradation by amylase activities (Lin et al., 2016). Finally, protein bodies in the endosperm are also implicated in chalkiness. Several studies have shown that chalky rice contains abnormal protein bodies in the endosperm that are large in size and accommodate more air spaces (Fukuda et al., 2011; Nagamine et al., 2011; Ren et al., 2014).

In this study, rice genotypes consisting of well‐known chalky varieties and low‐chalk cultivars were subjected to gene expression analysis as well as the analysis of grain physical characteristics, starch components, and the granule morphology. The expression patterns of *AGPL1*, *2*, *4*, *GBSSI*, and *SSIIA* was found to be genotype dependent and heat sensitive, which highlights the importance of coordinated starch biosynthesis during the critical stages of grain filling to produce properly filled, translucent (non‐chalky) rice grains. The disruption in the expression pattern of these starch biosynthesis genes by heat appears to be a part of the mechanism associated with the environment‐induced chalkiness.

## 2 MATERIALS AND METHODS

### 2.1 Plant materials

Six genotypes, ZHE 733, Nagina 22, Nipponbare, Taggart, Diamond, and LaGrue, representing *indica*, *aus*, *or japonica* subspecies were used in this study. These genotypes included three cultivars (Taggart, Diamond, and LaGrue) developed at Arkansas Rice Research Center. Three replications of each genotype were planted in July 2019 in the greenhouse. When plants were at R0 or R1 stage (Moldenhauer et al., 2018), they were transferred to growth chambers set at 30°C day/22°C night (normal) or at 30°C day/28°C night (HNT) with nighttime starting at 8 p.m. and ending at 6 a.m. Relative humidity and lighting conditions were uniform for the two set‐ups. The rice plant culms entering the reproductive stage were tagged and used as the source of samples for different stages, namely before panicle emergence (BP, also called R2 according to Moldenhauer et al., 2018), early flowering/after panicle emergence (AP), 5 days after flowering (DAF), 10, 15, and 20 DAF. For the granular, physical, and chemical properties, grains from the second panicle were collected at 25 DAF and dried at room temperature for 2 weeks to a moisture content of about ~12%. For gene expression analysis, spikelets from three biological replicates for each genotype/treatment were collected and immediately frozen in liquid nitrogen and stored at −80°C.

### 2.2 Grain physical property

Grains collected at 25 DAF were dried under room temperature for 2 weeks after harvesting, prior to the observation for chalkiness. Chalkiness was measured using WinSEEDLE^TM^ with 150 grains for each genotype. The percentage of chalky grains and the average chalk size per grain were taken as measurements of chalkiness.

### 2.3 Gene expression from databases

Heatmaps were generated to select the appropriate genes of the starch biosynthesis pathway. The Rice Expression Profile Database (Sato et al., 2011) was used under the category datasets and gene expression profile at different ripening stages (7, 10, 14, 21, 28, and 42 DAF) relevant to the stages selected for gene expression analysis by quantitative PCR.

### 2.4 Transcript levels

Total RNA was isolated using Trizol (Invitrogen Inc.) and quantified using Nano‐drop 2000 (Thermo‐Fisher Inc). Two micrograms of total RNA were treated with RQ1‐RNAse free DNase (Thermofisher Inc.), and one microgram of the DNase‐treated RNA was used for cDNA synthesis using PrimeScript RT reagent kit (Takara Bio). The expression analysis was performed using TB green Premix Ex Taq II (Takara Bio) on Bio‐Rad CFX 96 C1000 with following conditions: 95°C for 30 s. and 40 cycles of 95°C for 5 s +60°C for 30 s. The product specificity was verified by the melt curve analysis. The Ct values of genes‐of‐interest were normalized against 7Ubiquitin fused protein (*7UBIQ*) as the reference gene. Primers used in the study are given in Table S1.

### 2.5 Starch granule morphology

Grains were split in two using a microtome for the cross‐section perspective. Cross‐section of the grains were viewed under a Philips/Fei XL‐30 environmental scanning electron microscope with the settings, Acc V. of 10 kV, 2000× magnification, 3.0 spot and 10 μm bar. The surfaces of whole grains were captured both in low and high magnifications. Captured images were adjusted to the brightness of 20, color balance of R (0), G (0), and B (−20), and gamma correction of 1.00.

### 2.6 Component analysis

Grains harvested at 25 DAF were dried at room temperature for 2 weeks (~12% moisture content), ground to fine powder in liquid N_2_ for the determination of soluble protein content or in a cyclone milling machine for amylose and amylopectin content. The amylose and amylopectin content of grains were determined using the Megazyme amylose/amylopectin assay (K‐AMYL) following the manufacturer's method. Quantification of the soluble fractions of protein was done using 50 mg of grain powder in 1 ml of TE buffer pH 8.0 using a Bradford assay against a standard curve of BSA (0, 2.5, 5.0, 7.5, and 10 µg/ml). Absorbance was read at 595 nm in a Bio‐Rad SmartSpec 3000 spectrophotometer.

### 2.7 Data analysis

The experiment for soluble protein, amylose, and amylopectin contents were conducted in a completely randomized design with three independent replications under six cultivars having a total sample size of 18. Data were subjected to arcsine transformation and one‐way ANOVA. To determine the significant differences in the amylose and amylopectin content and protein concentration, Tukey's multiple comparison test was used to compare the genotypes under normal condition, and Student's *t*‐test for pairwise comparison in the normal and heat conditions. All statistical analyses were performed in SAS statistical software (version 9.4, SAS Institute Inc.) and results are presented in Tables S2–S6.

## 3 RESULTS

### 3.1 Expression patterns of starch biosynthesis genes

**FIGURE 1:**
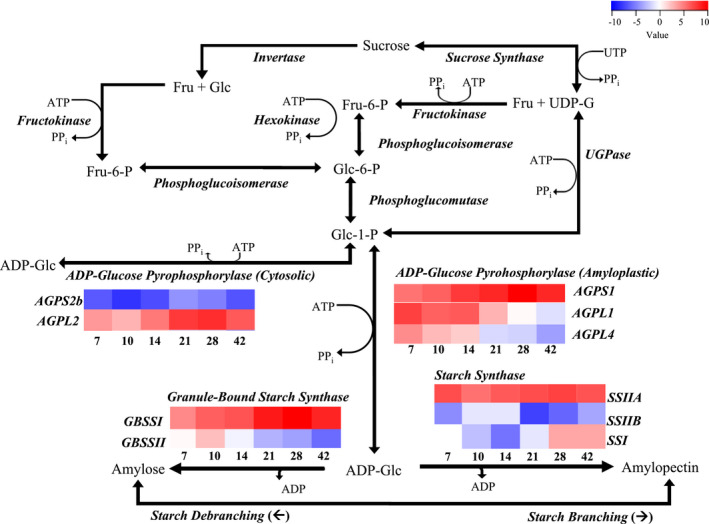
Illustration of the starch biosynthesis pathway. Flow of substrates and products are indicated by the arrows. Genes coding for the enzyme catalyzing a reaction are in bold and italics. Heatmaps based on the RiceXPro database (Sato et al. 2011) for the three genes in the two ultimate pathways (*ADP‐glucose pyrophospharalase*, *granule bound starch synthase*, and *starch synthase*) are placed under the gene name with the specific isoform or subunit indicated. Columns in the heatmap indicate endosperms from stages 7 to 42 DAF

The heatmap based on RiceXPro database showed genes encoding the subunits of rice amyloplastic AGPase are expressed differentially. *AGPS1* is somewhat consistent, while *AGPL1* and *AGPL4*, are upregulated early at 7–14 DAF followed by gradual decline in the subsequent stages with *AGPL4* expressed at relatively lower levels (Figure 1). *AGPL2* that encodes unique subunit of the cytosolic AGPase is expressed throughout the grain filling stages but shows upregulation in the mid grain filling stages (21–28 DAF) (Figure 1). The resulting cytosolic ADP‐glucose passes through the adenylate transporter, BRITTLE1 (BT1), to enter the amyloplast for starch biosynthesis (Cakir et al., 2016). AGPS2b subunit of the cytosolic AGPase, on the other hand, was expressed at much lower levels throughout the grain filling stages (Figure 1). AGPS2b forms heterotetramer with AGPL2 to form the cytosolic AGPase. For gene expression analysis of the cytosolic *AGPase*, *AGPL2* subunit gene was selected. Similarly, heatmap of GBSS showed that *GBSSII* is expressed at much lower levels and downregulated during advancing stages of grain filling (14 DAF onwards), while *GBSSI* is consistently expressed. Next, *SSI* is downregulated between 7 and 21 DAF and *SSIIB* is downregulated throughout (7–42 DAF), while *SSIIA* is consistently expressed (Figure 1). Therefore, *AGPL1*, *AGPL2*, *AGPL4*, *GBSSI*, *SSIIA*, and *BT1* were selected for gene expression analysis.

### 3.2 Grain chalkiness in different genotypes

**FIGURE 2:**
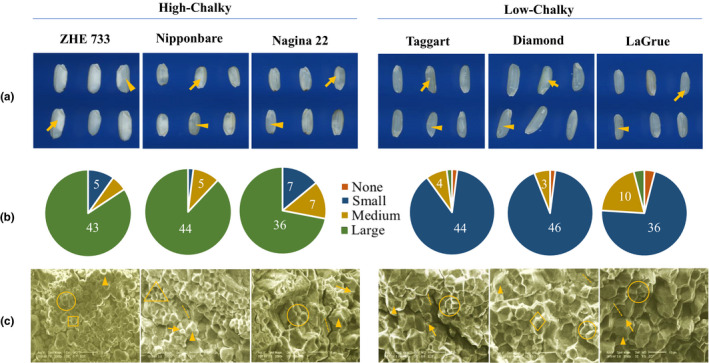
Physical characteristics of rice grains and granule morphology of different genotypes. (a) Grain morphology in WinSEEDLE. Arrow points to the chalky area and arrowhead indicates a translucent portion of a grain. (b) Distribution of grains with different chalkiness. Chalk sizes were classified relative to the area of the grain. None (no chalk), small (less than 10%), medium (11%–20%). Numbers are frequency counts from 50 grains observed. (c) Morphology of the grain transverse cross‐section under scanning electron microscopy with a scale bar of 10 μm. Arrowhead indicates the surface of amyloplast. Rings show polyhedral granules inside split amyloplast. Square indicates simple spherical granule. Triangle indicates simple polyhedral granules. Arrow points to protein bodies. Dashed line indicates the cracks from cross‐sectioning the samples. Diamonds show micropores on the surface of amyloplast

The six genotypes used in this study were found to have different levels of chalkiness based on which they were classified as high or low chalky. High chalky lines, ZHE 733, Nipponbare, and Nagina 22, contain large opaque areas, while the three low chalky cultivars, Taggart, Diamond, and LaGrue contain no chalk or small chalky areas (Figure 2a). Furthermore, in high‐chalky lines, chalk was observed in all grains, with the majority (average of 82%) showing large chalk (>20% of grain size), in addition to small (<10% of grain size) and medium (11%–20% of grain size) chalk. On the other hand, in low‐chalky lines, small chalk was found in the majority of the grains (average of 84%) with a small percentage (2%–4%) showing no chalk (Figure 2b). Among these, LaGrue was found to contain more chalk (medium sizes) than Taggart or Diamond.

### 3.3 Starch granule morphology

Scanning electron microscope images showed differences in the starch granule morphology between high chalky and low chalky lines. Cross‐section of the grains of high chalky lines revealed simple granules of spherical or polyhedral shape (Figure 2c). The high chalky lines, Nipponbare and Nagina 22, show polyhedral granules of simple and compound types; however, the sizes of the compound granules are not uniform. Protein bodies as defined by Kasem et al. (2011) are also observed in between and at the surface of the amyloplasts of ZHE 733 and Nipponbare (Figure 2c). Grains from low chalky lines showed compact granule structure of compound type. Moreover, the granules are homogeneous with smaller protein bodies. However, micropores were observed on the amyloplastic surface of Diamond (Figure 2c).

### 3.4 Expression patterns of AGPL2, AGPL4, GBSSI, and SSIIA

**FIGURE 3:**
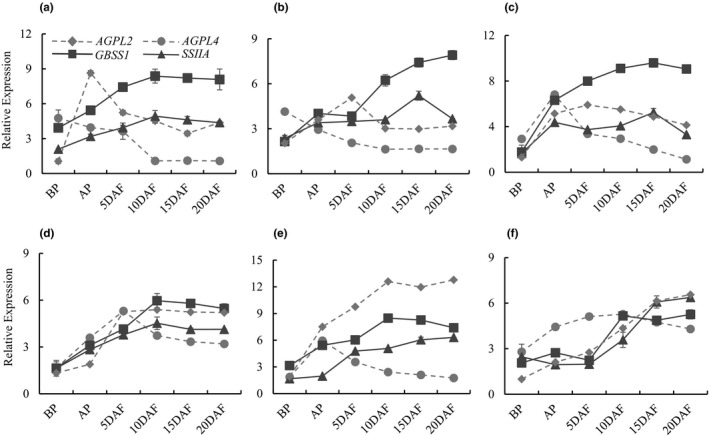
Gene expression patterns in the spikelets of different genotypes through early reproductive stages. Expression patterns of *AGPL2*, *AGPL4*, *GBSSI*, and *SSIIA* during reproductive phases in rice with reference to the *7UBIQ* expression. Each point is the mean of three biological replicates, and the error bars indicate standard error of the mean. (a) ZHE 733, (b) Nipponbare, (c) Nagina 22, (d) Taggart, (e) Diamond, and (f) LaGrue

In high chalky lines, AGPase subunit genes are expressed at higher levels during early reproductive stages (BP, AP, or 5 DAF) followed by gradual decline in the subsequent stages (5–20 DAF) when the upregulation of starch synthases occurs (Figure 3a–c). *GBSS1* showed rapid increase during this phase, and *SSIIA* showed a relatively lower but consistent expression. In ZHE 733 and Nipponbare, *AGPL2* and *AGPL4* expression declines during early grain filling stages (Figure 3a,b), and in Nagina 22, although the peak‐expression of these genes coincides with the upregulation of *GBSSI* and *SSIIA*, expression of *SSIIA* flattens and then appears to decline at 20 DAF (Figure 3c). The three low chalky lines, on the other hand, showed coordinated upregulation of one or both of *AGPL2* and *AGPL4* along with *GBSS1* and *SSIIA* (Figure 3d–f). Cultivar Taggart showed a tight co‐expression pattern of *AGPL2* and *AGPL4* with the starch synthase genes, *GBSS1*, and *SSIIA* (Figure 3d). In Diamond, while *AGPL4* expression gradually declined, during early grain filling stages (5–20 DAF), a sharp increase in *AGPL2* expression occurred in a pattern similar to that of *GBSSI* and *SSIIA* (Figure 3e). Finally, in LaGrue, *AGPL2*, *GBSSI*, and *SSIIA* were co‐expressed and markedly upregulated during early grain filling stages (5–20 DAF), while *AGLP4* was expressed early and remained consistent through early grain filling stages (Figure 3f).

### 3.5 Component analysis

There is a significant difference in the amylose content among high and low‐chalky cultivars (ANOVA, *α* = 0.05, *df* = 5, and *p* < 0.001), where ZHE 733 was the highest (Tukey's test, *α* = 0.05, *df* = 12 and *p* < 0.001). Within the high chalky group, the *indica* rice ZHE 733, that shows simple and spherical granules (Figure 2c), contained significantly higher amylose fraction. Low chalky cultivars, on the other hand, were found to contain significantly higher amylopectin content (Table 1). Regardless of the chalkiness, amylopectin is higher than amylose in all lines. Variation in the soluble protein content of the endosperm was also observed in the present study (ANOVA, *α* = 0.05, *df* = 5, and *p* = 0.0079). Nipponbare and Nagina 22, the two chalky lines, were found to have highest soluble proteins compared to other genotypes (Tukey's test, *α* = 0.05, *df* = 12, *p* < 0.001). The high chalky line, ZHE 733, on the other hand, showed a similar level of the soluble protein content as the three low chalky cultivars (Table 1).

**TABLE 1:** Amylose/Amylopectin and soluble protein content of high and low‐chalky cultivars/lines in rice under normal condition

Component	High‐chalky			Low‐chalky		
	ZHE 733	Nipponbare	Nagina 22	Taggart	Diamond	LaGrue
Amylose** (%)	32.12^a^	18.35^b^	19.03^b^	11.56^c^	12.48^c^	6.44^d^
Amylopectin** (%)	67.88^d^	81.65^c^	80.97^c^	88.44^b^	87.52^b^	93.56^a^
Soluble Protein** (mg/ml)	0.012^d^	0.025^ab^	0.022^bc^	0.013^d^	0.015^cd^	0.014^d^

Note

Means within each row having the same letter are not significantly different according to Tukey's multiple comparison at *α* = 0.05.

Analyses of variances (ANOVA) summaries are in Tables S2–S4.

Data were transformed using arcsine transformation.

** Significant for cultivar/line as source of variation at *p* = 0.01.

### 3.6 Effect of HNT on the gene expression patterns

All three large subunit genes of endosperm‐localized AGPase (*AGPL1*, *AGPL2*, and *AGPL4*), ADP‐glucose transporter gene (*BT1*), and starch synthase genes (*GBSS1* and *SSIIA*) were subjected to gene expression analysis during early reproductive stages in Diamond, LaGrue, and ZHE 733 grown under HNT.

**FIGURE 4:**
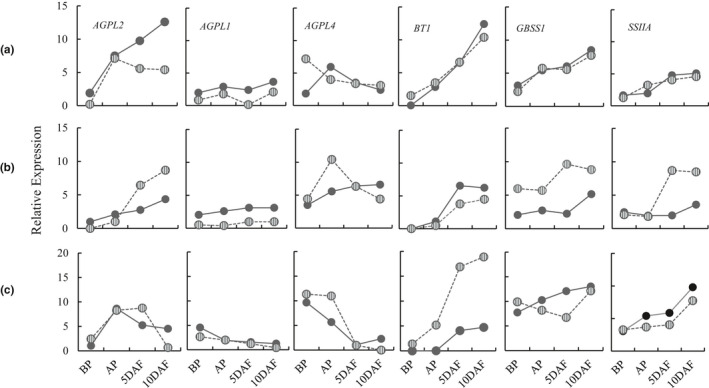
Effect of high nighttime temperature (HNT) on gene expression. Relative expression of *AGPL1*, *AGPL2*, *AGPL4*, *BT1‐1*, *GBSS1*, and *SSIIA* in the normal (solid lines) and HNT conditions (dashed lines) in the spikelets of rice at different reproductive stages with reference to the *7UBIQ* expression. Each point is the mean of three biological replicates. (a) Diamond, (b) LaGrue, and (c) ZHE 733

In Diamond, the most striking alteration occurred in AGPase subunit genes, while *BT1*, *GBSS1*, and *SSIIA* were mostly stable during early reproductive stages (Figure 4a). The cytosolic AGPase subunit gene *AGPL2* was markedly suppressed during 5–10 DAF and the amyloplastic AGPase subunit gene, *AGPL1*, was suppressed throughout the reproductive stages. In LaGrue, HNT‐treatment‐induced perturbation of all genes during the early reproductive stages. While, cytosolic AGPase subunit gene *AGPL2* was ≥2× upregulated during 5–10 DAF, amyloplastic AGPase subunit gene *AGPL1* was suppressed, and *AGPL4* spiked at the AP stage. *BT1* also showed minor suppression under HNT. However, *GBSSI* and *SSIIA* were markedly elevated by HNT in LaGrue (Figure 4b). Finally, in ZHE 733, *AGPL1*, *AGPL2*, and *AGPL4* were all suppressed at 10 DAF, although some spike in activity was observed for *AGPL2* and *AGPL4* in the preceding stages. *BT1*, on the other hand, was 4–5× upregulated, and starch synthase genes, *GBSS1* and *SSIIA*, showed minor suppression during early grain filling stages (Figure 4c).

**FIGURE 5:**
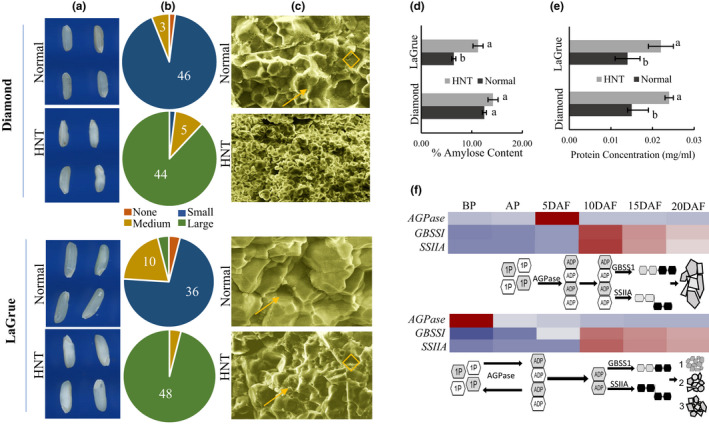
Physicochemical analysis of grains under normal and high nighttime temperature (HNT) conditions. (a, b) Grain images captured using WinSEEDLE^TM^ and distribution of chalk sizes in 50 grains. (c) Granule morphology under scanning electron microscope of Diamond and LaGrue with a scale bar of 10 μm. Arrows indicate protein bodies and diamonds indicate micropores. (d, e) Amylose and soluble protein content in Diamond and LaGrue in normal and HNT condition. Error bars represent standard errors within a group and bars with the same letter are not significantly different according to *t*‐test at *α* = 0.05. (f) Proposed mechanism of grain chalkiness. Coordinated (top) and uncoordinated (bottom) expression of *AGPase* subunit genes and starch synthase genes, *GBSSI* and *SSIIA*, through early reproductive phases. In the coordinated expression pattern, AGPL1 and 2, the regulatory subunits of the tetrameric AGPase, and monomeric GBSS1 and SSIIA are upregulated in quick succession through early grain filling stages. As a result, the conversion of glucose‐1‐phosphate (1P) to ADP‐glucose (ADP) occurs in a timely manner to efficiently synthesize amylose and amylopectin. As a result, large polyhedral granules are produced that pack tightly in the grains. In the uncoordinated expression pattern, early upregulation and/or subsequent suppression of AGPase subunit genes creates a temporal gap between the activation of AGPase and that of GBSSI and SSIIA, leading to non‐utilization of ADP and its reversal to 1P. As a result, suboptimal starch biosynthesis occurs leading to the formation of smaller granules of spherical (1) or polyhedral shapes (2, 3) accommodating airspaces and large protein bodies (3) that appear as grain chalk. The heatmap represents developmental expression pattern of the high‐chalky and low‐chalky line through reproductive stages: before panicle emergence (BP), after panicle emergence (AP), and 5–20 days after fertilization (DAF). Linear chain of hexagons indicates amylose (*α*1→4 glycosidase linkage) and branched chain indicates amylopectin (*α*1→4 and 1→6 glycosidase linkage)

Next, physical, granular, and chemical properties of Diamond and LaGrue grains were analyzed. The chalk distribution and grain morphology in the two cultivars completely changed upon HNT treatment. In the normal condition, these cultivars produced translucent grains with mostly small chalky areas; however, under HNT, both cultivars developed large chalky areas (Figure 5a,b). However, the granule morphology under HNT was different in the two cultivars. HNT‐Diamond developed simple spherical granules that resembled the granule morphology of normal‐ZHE 733 (Figures 2c and 5c). HNT‐LaGrue, on the other hand, maintained compound polyhedral granules but showed abundant shreds and micropores on the granule surfaces (Figure 5c).

Finally, amylose and soluble protein contents were determined under normal and HNT conditions. In Diamond, no significant difference in the amylose content (*t*‐test, *α* = 0.05, *df* = 5 and *p* = 0.0757) was observed between the two conditions, but in LaGrue an increase in the amylose content (*t*‐test, *α* = 0.05, *df* = 5 and *p* = 0.0008) was observed in the HNT grains (Figure 5d). However, the soluble protein content in Diamond (*t*‐test, *α* = 0.05, *df* = 5 and *p* = 0.0005) and LaGrue (*t*‐test, *α* = 0.05, *df* = 5 and *p* = 0.0004) was significantly higher under HNT compared to normal condition (Figure 5e).

## 4 DISCUSSION

### 4.1 Dynamics of starch biosynthesis genes

ADP‐glucose pyrophosphorylase, GBSS, and SS participate in the key steps of starch biosynthesis process. To analyze the cytosolic AGPase, that contributes to the bulk of AGPase activity in rice (Sikka et al., 2001), *AGLP2*, the large subunit gene unique to cytosolic AGPase was selected. We also analyzed the amyloplastic AGPase through expression analysis of its large subunit genes, *AGPL1* and/or *AGPL4*, as amyloplastic AGPase is considered critical for the normal levels of storage starch in the grains (Kawagoe et al., 2005; Lee et al., 2007; Sun et al., 2015). Of the GBSS and SS isoforms, *GBSS1* and *SSIIA* were selected based on their steady expression through grain filling stages (Figure 1; Hirose & Terao, 2004), and their roles in controlling amylose and amylopectin content, respectively, in rice (Dobo et al., 2010; Liu et al., 2014; Miura et al., 2018; Nakamura et al., 2005). The interpretation of starch biosynthesis process in this study was based solely on the gene expression analysis. Although, posttranscriptional and posttranslational controls of starch biosynthesis cannot be ignored (Smith et al., 2004; Tetlow et al., 2004; Wang et al., 1995), direct correlation of mRNA abundance and enzyme activity or protein abundance for AGPase, GBSS, and SS in rice and maize (Devi et al., 2010; Ponnala et al., 2014) supports the value gene expression analysis in gaining insights into the regulation of starch biosynthesis process.

Six rice genotypes were classified as high chalky or low chalky based on their grain chalkiness and granule morphology (Figure 2). Gene expression analysis showed coordination between AGPase subunit genes, *AGPL2* and *AGPL4*, and the two starch synthase genes, *GBSSI* and *SSIIA*, among the low chalky lines, presumably allowing efficient conversion of ADP‐glucose to amylose and amylopectin during early grain filling stages. However, an uncoordinated process was evident in high chalky lines that showed a gradual decline in the expression of *AGPL2* and *AGPL4* or peak expression before endosperm development (Figure 3). This uncoordinated process may result in futile ADP‐glucose reaction, and a limited pool of the metabolite during the critical stages of grain filling (5–20 DAF). Furthermore, since ADP‐glucose is unstable in the cell (Baroja‐Fernandez et al., 2004; Muñoz et al., 2005), its production before endosperm development (BP or AP stages) would likely result in its reversal to glucose‐1‐phosphate. Concurring with this hypothesis, a previous study showed that uncoordinated expression of AGPase subunit genes (*AGPL3*, *AGPL4*, and *AGPLS2*), subsequent to the peak expression of *GBSS* and *SS*, was associated with the inferior grains (Sun et al., 2015).

### 4.2 Correlation of granule morphology with gene expression patterns

High chalky lines showed simple granules of small sizes or compound granules of variable sizes (Figure 2c), suggesting inefficient or irregular starch biosynthesis leading to restricted enlargement of the granules (Kawagoe et al., 2005; Lisle et al., 2000). This hypothesis is supported by the expression analysis that showed temporal gap between the expression of AGPase subunit genes (*AGPL2* and *AGPL4*) and that of starch synthases (*GBSSI* and *SSIIA*; Figure 3a–c). The resulting insufficiency of ADP‐glucose at the early grain filling stages presumably leads to the simple or heterogeneous granules that accommodate airspaces. In support of this, Cakir et al., (2016) showed that loss of ADP‐glucose transporter gene, *BT1*, compromises carbon flux into amyloplast and leads to inefficient starch biosynthesis and formation of smaller, spherical starch granules. Low chalky lines, on the other hand, showed compound granules of larger sizes. This kind of granule is produced when substrates and enzymes are sufficiently available at the critical stages of grain filling (Toyosawa et al., 2016). These conditions appear to have been fulfilled in the low chalky cultivars, as indicated by the coordination in the expression pattern of *AGPL2*, *AGPL4*, *GBSSI*, and *SSIIA* (Figure 3d–f). However, as observed in the Diamond (Figure 2c), compound granules could later develop pits and micropores that contribute to chalkiness (Kaneko et al., 2016; Lin et al., 2016; Mitsui et al., 2016; Tsutsui et al., 2013). These micropores are considered the evidence of the enzymatic degradation of starch (Lin et al., 2014; Zakaria et al., 2002), a hypothesis supported by the genetic analysis that showed improvement in rice grain quality upon suppression of the α‐amylase genes (Hakata et al., 2012).

### 4.3 Starch components and their correlation with grain chalkiness

High amylose content has been correlated with heterogeneous granule formation and grain chalkiness (Man et al., 2014; Zheng et al., 2012). Accordingly, in this study, higher amylose content was observed in high chalky lines (Table 1). Shorter chains (*α*1–4) and reduced branching (*α*1–6) in the amylopectin could lead to smaller granules. This analogy is reinforced by observations that high amylose rice contains shorter α‐glucan chain lengths, and grains containing short chain amylopectin show higher chalkiness (Park et al., 2007; Patindol & Wang, 2003), possibly from loose packing of small polyhedral granules (Zhang et al., 2017). On the other hand, higher amylopectin was found in the low chalky lines, which corroborates with other studies that found the association of higher amylopectin with lower chalkiness (Inukai, 2017; Lin et al., 2016). The coordinated expression of *AGPase* subunit genes and *GBSSI* and *SSIIA* in the low‐chalky cultivars (Figure 3d–f), arguably favors efficient synthesis of amylopectin. However, all lines regardless of the chalk levels contain higher amylopectin than amylose (Table 1), pointing to the possible roles of SSI and the starch branching enzymes (SBE) in enhancing amylopectin levels in later stages of grain development. Accordingly, *SSI* and *SBEIIB* mutants of rice show decrease in amylopectin content (Abe et al., 2014; Sun et al., 2017). Finally, although genetic relation of protein content with the quality traits in rice has been described (Lin et al., 2016; Liu et al., 2011; Zheng et al., 2012), its correlation with grain chalkiness is not very clear. In this study, variation in the soluble protein content was observed (Table 1), but no clear correlation with grain chalkiness was observed.

### 4.4 Mechanisms of heat‐induced chalkiness

High nighttime temperature induces chalk formation along with the changes in the expression of starch biosynthesis genes (Dhatt et al., 2019; Mitsui et al., 2016; Nevame et al., 2018). In the present study, both downregulation and upregulation of individual genes was observed in Diamond, LaGrue, and ZHE 733 (Figure 4a–c). This differential perturbation suggests that the mechanism of grain chalkiness could differ between genotypes. However, since ZHE 733 is chalky under normal condition, analysis of granule morphology and component analysis under HNT was done only in Diamond and LaGrue. An increase in amylose and soluble protein content was generally observed under HNT in both cultivars (Figure 5d–e). However, granule morphology under HNT was distinct between Diamond and LaGrue (Figure 5c).

In Diamond, the change in granule morphology to simple, spherical type, was accompanied by the suppression of AGPase subunit genes *AGPL1* and *AGPL2* (Figure 4a). Thus, HNT‐induced chalkiness in Diamond could be attributed to inefficient starch biosynthesis due to suppression of the first key step in starch biosynthesis. In LaGrue, starch biosynthesis is upregulated under HNT as indicated by the upregulation of *AGPL2*, *GBSSI*, and *SSIIA* (Figure 4b). Even though other genes are suppressed, the retention of compound granules in HNT‐LaGrue indicate that starch biosynthesis is not significantly compromised under HNT. Therefore, a distinct mechanism of HNT‐induced chalkiness is involved in LaGrue, possibly involving amylase attack in the late developmental stages as shreds and micropores on starch granules were evident on the surfaces of granules (Figure 5c). The formation of shreds and micropores has widely been implicated as the evidence of starch degradation through upregulation of α‐amylase genes (Iwasawa et al., 2009; Tsutsui et al., 2013; Yamakawa et al., 2007; Zakaria et al., 2002). Finally, heat‐induced chalk formation in rice is also accompanied with the increase in all classes of storage protein fractions, including albumins and globulins in the early phases of grain development (Lin et al., 2010). However, association of protein content and chalkiness is more complex and likely involves formation of protein bodies with reduced storage proteins accommodating more air pockets (Wada et al., 2018).

## 5 CONCLUSIONS

Efficient synthesis of starch through the coordinated expression of *AGPase*, *GBSS*, and *SS* is arguably the most important mechanism controlling granule morphology. In the coordinated expression pattern, *AGPase* is upregulated early in the reproductive phase, during the grain filling stages, to produce abundant pool of ADP‐glucose, which is quickly utilized by GBSS and SS to produce amylose and amylopectin in the amyloplast. This streamlined process leads to uniform polyhedral granules that pack tightly and produce non‐chalky grains (Figure 5f). However, when *AGPase* is upregulated before endosperm development or suppressed by heat treatment, only a limited pool of ADP‐glucose is presumably available for starch biosynthesis. This uncoordinated process could lead to inefficient starch biosynthesis, producing smaller granules of heterogeneous shapes (Figure 5f). These simple spherical or heterogeneous granules pack more loosely and accommodate air spaces observed as chalk in the mature grains.

## CONFLICT OF INTEREST

The authors have no conflict of interest.

## Supporting information

Table S1‐S6

## Data Availability

The data that support the findings of this study are openly available in the Box folder hosted by University of Arkansas at https://uark.box.com/s/55vfk9pndgf2pmrtj2vqpkr783o83r9b.

## References

[pei310054-bib-0001] Abe, N. , Asai, H. , Yago, H. , Oitome, N. F. , Itoh, R. , Crofts, N. , Nakamura, Y. , & Fujita, N. (2014). Relationships between starch synthase I and branching enzyme isozymes determined using double mutant rice lines. BMC Plant Biology, 14, 80. 10.1186/1471-2229-14-80 24670252 PMC3976638

[pei310054-bib-0002] Baroja‐Fernández, E. , Muñoz, F. J. , Zandueta‐Criado, A. , Morán‐Zorzano, M. T. , Viale, A. M. , Alonso‐Casajús, N. , & Pozueta‐Romero, J. (2004). Most of ADP x glucose linked to starch biosynthesis occurs outside the chloroplast in source leaves. Proceedings of the National Academy of Sciences of the United States of America, 101(35), 13080–13085. 10.1073/pnas.0402883101 15326306 PMC516520

[pei310054-bib-0003] Cakir, B. , Shiraishi, S. , Tuncel, A. , Matsusaka, H. , Satoh, R. , Singh, S. , Crofts, N. , Hosaka, Y. , Fujita, N. , Hwang, S. K. , Satoh, H. , & Okita, T. W. (2016). Analysis of the rice ADP‐Glucose Transporter (OsBT1) indicates the presence of regulatory processes in the amyloplast stroma that control ADP‐Glucose flux into starch. Plant Physiology, 170(3), 1271–1283. 10.1104/pp.15.01911 26754668 PMC4775147

[pei310054-bib-0004] Devi, T. A. , Sarla, N. , Siddiq, E. A. , & Sirdeshmukh, R. (2010). Activity and expression of adenosine diphosphate glucose pyrophosphorylase in developing rice grains: Varietal differences and implications on grain filling. Plant Science, 178(2), 123–129. 10.1016/j.plantsci.2009.10.008

[pei310054-bib-0005] Dhatt, B. K. , Abshire, N. , Paul, P. , Hasanthika, K. , Sandhu, J. , Zhang, Q. , Obata, T. , & Walia, H. (2019). Metabolic dynamics of developing rice seeds under high night‐time temperature stress. Frontiers in Plant Science, 10, 1443. 10.3389/fpls.2019.01443 31781147 PMC6857699

[pei310054-bib-0006] Dobo, M. , Ayres, N. , Walker, G. , & Park, W. (2010). Polymorphism in the GBSS gene affects amylose content in US and European rice germplasm. Journal of Cereal Science, 52(3), 450–456. 10.1016/j.jcs.2010.07.010

[pei310054-bib-0008] Feng, F. , Li, Y. , Qin, X. , Liao, Y. , & Siddique, K. (2017). Changes in rice grain quality of indica and japonica type varieties released in China from 2000 to 2014. Frontiers in Plant Science, 8, 1863. 10.3389/fpls.2017.01863 29163589 PMC5671604

[pei310054-bib-0009] Fitzgerald, M. A. , & Resurreccion, A. P. (2009). Maintaining the yield of edible rice in a warming world. Functional Plant Biology, 36(1), 1037–1045. 10.1071/FP09055 32688715

[pei310054-bib-0010] Fukuda, M. , Satoh‐Cruz, M. , Wen, L. , Crofts, A. J. , Sugino, A. , Washida, H. , Okita, T. W. , Ogawa, M. , Kawagoe, Y. , Maeshima, M. , & Kumamaru, T. (2011). The small GTPase Rab5a is essential for intracellular transport of proglutelin from the Golgi apparatus to the protein storage vacuole and endosomal membrane organization in developing rice endosperm. Plant Physiology, 157(2), 632–644. 10.1104/pp.111.180505 21825104 PMC3192576

[pei310054-bib-0011] Hakata, M. , Kuroda, M. , Miyashita, T. , Yamaguchi, T. , Kojima, M. , Sakakibara, H. , Mitsui, T. , & Yamakawa, H. (2012). Suppression of α‐amylase genes improves quality of rice grain ripened under high temperature. Plant Biotechnology Journal, 10(9), 1110–1117. 10.1111/j.1467-7652.2012.00741.x 22967050

[pei310054-bib-0012] Hirose, T. , & Terao, T. (2004). A comprehensive expression analysis of the starch synthase gene family in rice (*Oryza* *sativa* L.). Planta, 220(1), 9–16. 10.1007/s00425-004-1314-6 15232694

[pei310054-bib-0013] Inukai, T. (2017). Differential regulation of starch‐synthetic gene expression in endosperm between indica and japonica rice cultivars. Rice, 10(1), 7. 10.1186/s12284-017-0146-5 28243987 PMC5328889

[pei310054-bib-0014] Iwasawa, N. , Umemoto, T. , Hiratsuka, M. , Nitta, Y. , Matsuda, T. , & Kondo, M. (2009). Structural characters of milky‐white rice grains caused by high temperature and shading during grain‐filling. Japanese Journal of Crop Science, 78, 322–323. 10.14829/jcsproc.227.0.330.0

[pei310054-bib-0015] Jagadish, S. V. K. , Murty, M. V. R. , & Quick, W. P. (2015). Rice responses to rising temperatures. Plant, Cell & Environment, 38, 1686–1698. 10.1111/pce.12430 25142172

[pei310054-bib-0016] Kaneko, K. , Sasaki, M. , Kuribayashi, N. , Suzuki, H. , Sasuga, Y. , Shiraya, T. , Inomata, T. , Itoh, K. , Baslam, M. , & Mitsui, T. (2016). Proteomic and glycomic characterization of rice chalky grains produced under moderate and high‐temperature conditions in field system. Rice, 9(1), 26. 10.1186/s12284-016-0100-y 27246013 PMC4887401

[pei310054-bib-0017] Kasem, S. , Waters, D. , Rice, N. , Shapter, F. , & Henry, R. (2011). The endosperm morphology of rice and its wild relatives as observed by scanning electron microscopy. Rice, 4, 12–20. 10.1007/s12284-011-9060-4

[pei310054-bib-0018] Kawagoe, Y. , Kubo, A. , Satoh, H. , Takaiwa, F. , & Nakamura, Y. (2005). Roles of isoamylase and ADP‐glucose pyrophosphorylase in starch granule synthesis in rice endosperm. The Plant Journal, 42, 164–174. 10.1111/j.1365-313X.2005.02367.x 15807780

[pei310054-bib-0019] Kim, K. S. , Kang, H. J. , Hwang, I. K. , Hwang, H. G. , Kim, T. Y. , & Choi, H. C. (2004). Comparative ultrastructure of Ilpumbyeo, a high‐quality japonica rice, and its mutant, Suweon 464: Scanning and transmission electron microscopy studies. Journal of Agricultural and Food Chemistry, 52(12), 3876–3883. 10.1021/jf049767r 15186110

[pei310054-bib-0020] Kusano, M. , Fukushima, A. , Fujita, N. , Okazaki, Y. , Kobayashi, M. , Oitome, N. , Ebana, K. , & Saito, K. (2012). Deciphering starch quality of rice kernels using metabolite profiling and pedigree network analysis. Molecular Plant, 5, 442–451. 10.1093/mp/ssr101 22180466

[pei310054-bib-0021] Lanning, S. , Siebenmorgen, T. , Counce, P. , Ambardekar, A. , & Mauromoustakos, A. (2011). Extreme nighttime air temperatures in 2010 impact rice chalkiness and milling quality. Field Crops Research, 124, 132–136. 10.1016/j.fcr.2011.06.012

[pei310054-bib-0022] Lee, S. K. , Hwang, S. K. , Han, M. , Eom, J. S. , Kang, H. G. , Han, Y. , Choi, S. B. , Cho, M. H. , Bhoo, S. H. , An, G. , Hahn, T. R. , Okita, T. W. , & Jeon, J. S. (2007). Identification of the ADP‐glucose pyrophosphorylase isoforms essential for starch synthesis in the leaf and seed endosperm of rice (*Oryza* *sativa* L.). Plant Molecular Biology, 65(4), 531–546. 10.1007/s11103-007-9153-z 17406793

[pei310054-bib-0023] Lin, C. J. , Li, C. Y. , Lin, S. K. , Yang, F. H. , Huang, J. J. , Liu, Y. H. , & Lur, H. S. (2010). Influence of high temperature during grain filling on the accumulation of storage proteins and grain quality in rice (*Oryza* *sativa* L.). Journal of Agricultural and Food Chemistry, 58(19), 10545–10552. 10.1021/jf101575j 20839801

[pei310054-bib-0024] Lin, Z. , Zheng, D. , Zhang, X. , Wang, Z. , Lei, J. , Liu, Z. , Li, G. , Wang, S. , & Ding, Y. (2016). Chalky part differs in chemical composition from translucent part of japonica rice grains as revealed by a notched‐belly mutant with white‐belly. Journal of the Science of Food and Agriculture, 96(11), 3937–3943. 10.1002/jsfa.7793 27166835 PMC5089642

[pei310054-bib-0025] Lisle, A. J. , Martin, M. , & Fitzgerald, M. A. (2000). Chalky and translucent rice grains differ in starch composition and structure and cooking properties. Cereal Chemistry, 77(5), 627–632. 10.1094/CCHEM.2000.77.5.627

[pei310054-bib-0026] Liu, D. , Wang, W. , & Cai, X. (2014). Modulation of amylose content by structure‐based modification of *OsGBSS1* activity in rice (*Oryza* *sativa* L.). Plant Biotechnology Journal, 12(9), 1297–1307. 10.1111/pbi.12228 25052102

[pei310054-bib-0027] Liu, X. , Guo, T. , Wan, X. , Wang, H. , Zhu, M. , Li, A. , Su, N. , Shen, Y. , Mao, B. , Zhai, H. , Mao, L. , & Wan, J. (2010). Transcriptome analysis of grain‐filling caryopses reveals involvement of multiple regulatory pathways in chalky grain formation in rice. BMC Genomics, 11, 730. 10.1186/1471-2164-11-730 21192807 PMC3023816

[pei310054-bib-0028] Liu, X. , Wan, X. , Ma, X. , & Wan, J. (2011). Dissecting the genetic basis for the effect of rice chalkiness, amylose content, protein content, and rapid viscosity analyzer profile characteristics on the eating quality of cooked rice using the chromosome segment substitution line population across eight environments. Genome, 54(1), 64–80. 10.1139/G10-070 21217807

[pei310054-bib-0029] Lu, G. , Third, J. , & Müller, C. R. (2015). Discrete element models for non‐spherical particle systems: From theoretical developments to applications. Chemical Engineering Science, 127(4), 425–465. 10.1016/j.ces.2014.11.050

[pei310054-bib-0030] Man, J. , Lin, L. , Wang, Z. , Wang, Y. , Liu, Q. , & Wei, C. (2014). Different structures of heterogeneous starch granules from high‐amylose rice. Journal of Agricultural and Food Chemistry, 62(46), 11254–11263. 10.1021/jf503999r 25373551

[pei310054-bib-0031] Mitsui, T. , Yamakawa, H. , & Kobata, T. (2016). Molecular physiological aspects of chalking mechanism in rice grains under high‐temperature stress. Plant Production Science, 19(1), 22–29. 10.1080/1343943X.2015.1128112

[pei310054-bib-0032] Miura, S. , Crofts, N. , Saito, Y. , Hosaka, Y. , Oitome, N. F. , Watanabe, T. , Kumamaru, T. , & Fujita, N. (2018). Starch synthase IIa‐deficient mutant rice line produces endosperm starch with lower gelatinization temperature than japonica rice cultivars. Frontiers in Plant Science, 9, 645. 10.3389/fpls.2018.00645 29868097 PMC5962810

[pei310054-bib-0033] Moldenhauer, K. , Counce, P. , & Hardke, J. (2018). Rice growth and development. Rice Production Handbook, 192, 7–14.

[pei310054-bib-0034] Muñoz, F. J. , Baroja‐Fernández, E. , Morán‐Zorzano, M. T. , Viale, A. M. , Etxeberria, E. , Alonso‐Casajús, N. , & Pozueta‐Romero, J. (2005). Sucrose synthase controls both intracellular ADP glucose levels and transitory starch biosynthesis in source leaves. Plant and Cell Physiology, 46, 1366–1376. 10.1093/pcp/pci148 15951568

[pei310054-bib-0035] Nagamine, A. , Matsusaka, H. , Ushijima, T. , Kawagoe, Y. , Ogawa, M. , Okita, T. W. , & Kumamaru, T. (2011). A role for the cysteine‐rich 10 kDa prolamin in protein body I formation in rice. Plant and Cell Physiology, 52, 1003–1016. 10.1093/pcp/pcr053 21521743 PMC3110882

[pei310054-bib-0036] Nakamura, Y. , Francisco, P. , Hosaka, Y. , Sato, A. , Sawada, T. , Kubo, A. , & Fujita, N. (2005). Essential amino acid of starch synthase IIa differentiate amylopectin structure and starch quality between japonica and indica rice varieties. Plant Molecular Biology, 58, 213–227. 10.1007/s11103-005-6507-2 16027975

[pei310054-bib-0037] Nevame, A. , Emon, R. M. , Malek, M. D. , Hasan, M. , Alam, M. A. , Muharam, F. M. , Aslani, F. , Rafii, M. , & Ismail, M. (2018). Relationship between high temperature and formation of chalkiness and their effects on quality of rice. BioMed Research International, 2018, 1–18. 10.1155/2018/1653721 PMC6051336 30065932

[pei310054-bib-0038] Ohdan, T. , Francisco, P. B. , Sawada, T. , Hirose, T. , Terao, T. , Satoh, H. , & Nakamura, Y. (2005). Expression profiling of genes involved in starch synthesis in sink and source organs of rice. Journal of Experimental Botany, 56, 3229–3244. 10.1093/jxb/eri292 16275672

[pei310054-bib-0039] Park, I. , Ibanez, A. , Zhong, F. , & Shoemaker, C. (2007). Gelatinization and pasting properties of waxy and non‐waxy rice starches. Starch, 59(8), 388–396. 10.1002/star.200600570

[pei310054-bib-0040] Patindol, J. , & Wang, Y.‐J. (2003). Fine structures and physicochemical properties of starches from chalky and translucent rice kernels. Journal of Agricultural and Food Chemistry, 51(9), 2777–2784. 10.1021/jf026101t 12696972

[pei310054-bib-0041] Ponnala, L. , Wang, Y. , Sun, Q. , & van Wijk, K. J. (2014). Correlation of mRNA and protein abundance in the developing maize leaf. Plant Journal, 78, 424–440. 10.1111/tpj.12482 24547885

[pei310054-bib-0042] Ren, Y. , Wang, Y. , Liu, F. , Zhou, K. , Ding, Y. , Zhou, F. , Wang, Y. , Liu, K. , Gan, L. , Ma, W. , Han, X. , Zhang, X. , Guo, X. , Wu, F. , Cheng, Z. , Wang, J. , Lei, C. , Lin, Q. , Jiang, L. , … Wan, J. (2014). Glutelin precursor accumulation3 encodes a regulator of post‐Golgi vesicular traffic essential for vacuolar protein sorting in rice endosperm. The Plant Cell, 26(1), 410–425. 10.1105/tpc.113.121376 24488962 PMC3963586

[pei310054-bib-0043] Sato, Y. , Antonio, B. A. , Namiki, N. , Takehisa, H. , Minami, H. , Kamatsuki, K. , Sugimoto, K. , Shimizu, Y. , Hirochika, H. , & Nagamura, Y. (2011). RiceXPro: A platform for monitoring gene expression in japonica rice grown under natural field conditions. Nucleic Acids Research, 39(Database issue), D1141–D1148. 10.1093/nar/gkq1085 21045061 PMC3013682

[pei310054-bib-0044] Sikka, V. K. , Choi, S. B. , Kavakli, I. H. , Sakulsingharoj, C. , Gupta, S. , Ito, H. , & Okita, T. W. (2001). Subcellular compartmentation and allosteric regulation of the rice endosperm ADP glucose pyrophosphorylase. Plant Science, 161, 461–468. 10.1016/S0168-9452(01)00431-9

[pei310054-bib-0045] Smith, S. M. , Fulton, D. C. , Chia, T. , Thorneycroft, D. , Chapple, A. , Dunstan, H. , Hylton, C. , Zeeman, S. C. , & Smith, A. M. (2004). Diurnal changes in the transcriptome encoding enzymes of starch metabolism provide evidence for both transcriptional and posttranscriptional regulation of starch metabolism in Arabidopsis leaves. Plant Physiology, 136(1), 2687–2699. 10.1104/pp.104.044347 15347792 PMC523333

[pei310054-bib-0046] Stark, D. M. , Timmerman, K. P. , Barry, G. F. , Preiss, J. , & Kishore, G. M. (1992). Regulation of the amount of starch in plant tissues by ADP glucose pyrophosphorylase. Science, 258(5080), 287–292. 10.1126/science.258.5080.287 17835129

[pei310054-bib-0047] Sun, H. , Peng, T. , Zhao, Y. , Du, Y. , Zhang, J. , Li, J. , Xin, Z. , & Zhao, Q. (2015). Dynamic analysis of gene expression in rice superior and inferior grains by RNA‐Seq. PLoS One, 10, e0137168. 10.1371/journal.pone.0137168 26355995 PMC4565701

[pei310054-bib-0048] Sun, Y. , Jiao, G. , Liu, Z. , Zhang, X. , Li, J. , Guo, X. , Du, W. , Du, J. , Francis, F. , Zhao, Y. , & Xia, L. (2017). Generation of high‐amylose rice through CRISPR/Cas9‐mediated targeted mutagenesis of starch branching enzymes. Frontiers in Plant Science, 8, 298. 10.3389/fpls.2017.00298 28326091 PMC5339335

[pei310054-bib-0049] Tetlow, I. , & Emes, M. (2017). Starch biosynthesis in the developing endosperms of grasses and cereals. Agronomy, 7, 81. 10.3390/agronomy7040081

[pei310054-bib-0050] Tetlow, I. J. , Morell, M. K. , & Emes, M. J. (2004). Recent developments in understanding the regulation of starch metabolism in higher plants. Journal of Experimental Botany, 55(406), 2131–2145. 10.1093/jxb/erh248 15361536

[pei310054-bib-0051] Toyosawa, Y. , Kawagoe, Y. , Matsushima, R. , Crofts, N. , Ogawa, M. , Fukuda, M. , Kumamaru, T. , Okazaki, Y. , Kusano, M. , Saito, K. , Toyooka, K. , Sato, M. , Ai, Y. , Jane, J. L. , Nakamura, Y. , & Fujita, N. (2016). Deficiency of starch synthase IIIa and IVb alters starch granule morphology from polyhedral to spherical in rice endosperm. Plant Physiology, 170(3), 1255–1270. 10.1104/pp.15.01232 26747287 PMC4775109

[pei310054-bib-0052] Tsutsui, K. , Kaneko, K. , Hanashiro, I. , Nishinari, K. , & Mitsui, T. (2013). Characteristics of opaque and translucent parts of high temperature stressed grains of rice. Journal of Applied Glycoscience, 60, 61–67. 10.5458/jag.jag.JAG-2012_014

[pei310054-bib-0053] Umemoto, T. , & Terashima, K. (2002). Research note: Activity of granule‐bound starch synthase is an important determinant of amylose content in rice endosperm. Functional Plant Biology, 29(9), 1121–1124. 10.1071/PP01145 32689564

[pei310054-bib-0054] Wada, H. , Hatakeyama, Y. , Onda, Y. , Nonami, H. , Nakashima, T. , Erra‐Balsells, R. , Morita, S. , Hiraoka, K. , Tanaka, F. , & Nakano, H. (2018). Multiple strategies for heat adaptation to prevent chalkiness in the rice endosperm. Journal of Experimental Botany, 70(4), 1299–1311. 10.1093/jxb/ery427 PMC6382329 30508115

[pei310054-bib-0055] Wang, E. , Wang, J. , Zhu, X. , Hao, W. , Wang, L. , Li, Q. , Zhang, L. , He, W. , Lu, B. , Lin, H. , Ma, H. , Zhang, G. , & He, Z. (2008). Control of rice grain‐filling and yield by a gene with a potential signature of domestication. Nature Genetics, 40(11), 1370–1374. 10.1038/ng.220 18820698

[pei310054-bib-0056] Wang, Z. Y. , Zheng, F. Q. , Shen, G. Z. , Gao, J. P. , Snustad, D. P. , Li, M. G. , Zhang, J. L. , & Hong, M. M. (1995). The amylose content in rice endosperm is related to the post‐transcriptional regulation of the waxy gene. The Plant Journal, 7(4), 613–622. 10.1046/j.1365-313x.1995.7040613.x 7742858

[pei310054-bib-0057] Xing, S. , Meng, X. , Zhou, L. , Mujahid, H. , Zhao, C. , Zhang, Y. , Wang, C. , & Peng, Z. (2016). Proteome profile of starch granules purified from rice (*Oryza* *sativa*) endosperm. PLoS One, 11(12), e0168467. 10.1371/journal.pone.0168467 27992503 PMC5167393

[pei310054-bib-0058] Xu, J. , Henry, A. , & Sreenivasulu, N. (2020). Rice yield formation under high day and night temperatures – A prerequisite to ensure future food security. Plant, Cell & Environment, 43, 1595–1608. 10.1111/pce.13748 32112422

[pei310054-bib-0059] Yamakawa, H. , Hirose, T. , Kuroda, M. , & Yamaguchi, T. (2007). Comprehensive expression profiling of rice grain filling‐related genes under high temperature using DNA microarray. Plant Physiology, 144(1), 258–277. 10.1104/pp.107.098665 17384160 PMC1913800

[pei310054-bib-0060] Zakaria, S. , Matsuda, T. , Tajima, S. , & Nitta, Y. (2002). Effect of high temperature at ripening stage on the reserve accumulation in seed in some rice cultivars. Plant Production Science, 5, 160–168. 10.1626/pps.5.160

[pei310054-bib-0061] Zhang, C. , Chen, S. , Ren, X. , Lu, Y. , Liu, D. , Cai, X. , Li, Q. , Gao, J. , & Liu, Q. (2017). Molecular structure and physicochemical properties of starches from rice with different amylose contents resulting from modification of *OsGBSSI* activity. Journal of Agricultural and Food Chemistry, 65(10), 2222–2232. 10.1021/acs.jafc.6b05448 28241110

[pei310054-bib-0062] Zheng, L. , Zhang, W. , Liu, S. , Chen, L. , Liu, X. , Xingang, C. , Ma, J. , Chen, W. , Zhao, Z. , Jiang, L. , & Wan, J. (2012). Genetic relationship between grain chalkiness, protein content, and paste viscosity properties in a backcross inbred population of rice. Journal of Cereal Science, 56, 153–160. 10.1016/j.jcs.2012.05.003

