# Supplementary material for: Genotype‐dependent and heat‐induced grain chalkiness in rice correlates with the expression patterns of starch biosynthesis genes
Source: Plant Environ Interact. 2021 Jun 15;2(4):165–76. doi: 10.1002/pei3.10054 (PMC10168090; doi:10.1002/pei3.10054)
Supplement: Supplementary file 1 — Table S1‐S6 [file PEI3-2-165-s001.docx]

| **Gene Name and ID** | **Primer Sequence (5’ – 3’)** |
| --- | --- |
| *AGPL1* (Os05g0580000) | Forward: GACTTGGAAGCAATGAAAGTGG |
|  | Reverse: ACTCCCATCGAAGCAATGTAG |
| *AGPL2* (Os01g0633100) | Forward: ACTGAGGAAGAGGTGCTTTG |
|  | Reverse: GAGGATTGTGTCCGAAGATGAG |
| *AGPL4* (Os07g0243200) | Forward: GCAAGAAGCAGAGAGACCATTA |
|  | Reverse: AACTGTACCATCTGGAATCACC |
| *GBSSI* (Os06g0133000) | Forward: GGTACTGGAAAGAAGAAGTTCG |
|  | Reverse: TCC GGC CAT GAT GAG ATG AGC AA |
| *SSIIA* (Os06g0229800) | Forward: GAGACGTACCGCAAGTACAA |
|  | Reverse: GACAAGGACCTCCTCGTAGA |
| *OSBT1* (Os02g0202400) | Forward: GGCATCTCCTTCATGTGCTA |
|  | Reverse: CAACCTTCTCCTTGATCTCCTC |
| *7UBIQUITIN* | Forward: TGGTCAGTAATCAGCCAGTTTG |
|  | Reverse: CAAATACTTGACGAACAGAGGC |

**Supplementary Table S1.** Primer sequences for qPCR

**Supplementary Table 2.** ANOVA for the amylose content across different cultivars under normal condition.

| Sources of Variation | Df | MS | F-Value | Pr>G |
| --- | --- | --- | --- | --- |
| Cultivar | 5 | 412.2830 | 52.99 | <.0001 |
| Error | 12 | 7.7803 |  |  |

**Supplementary Table 3.** ANOVA for the amylopectin content across different cultivars under normal condition.

| Sources of Variation | Df | MS | F-Value | Pr>G |
| --- | --- | --- | --- | --- |
| Cultivar | 5 | 8413.0830 | 52.99 | <.0001 |
| Error | 12 | 158.7673 |  |  |

**Supplementary Table 4.** ANOVA for the soluble protein content across different cultivars under normal condition.

| Sources of Variation | Df | MS | F-Value | Pr>G |
| --- | --- | --- | --- | --- |
| Cultivar | 5 | 0.00036502 | 5.40 | 0.0079 |
| Error | 12 | 0.00006760 |  |  |

**Supplementary Table 5.** T-Test analysis for the soluble protein content across different cultivars under normal and HNT condition.

| Cultivar | Df | t-Value | Pr>**\|t\|** |
| --- | --- | --- | --- |
| Diamond | 5 | 8.11 | 0.0005 |
| LaGrue | 5 | 8.49 | 0.0004 |

**Supplementary Table 6.** T-Test analysis for the amylose content across different cultivars under normal and HNT condition.

| Cultivar | Df | t-Value | Pr>**\|t\|** |
| --- | --- | --- | --- |
| Diamond | 5 | 1.81 | 0.0757 |
| LaGrue | 5 | 7.25 | 0.0008 |
